# Bilateral Variability of the Quadriceps Angle (Q angle) in an Adult Indian Population

**Published:** 2011

**Authors:** Raveendranath Raveendranath, Shankar Nachiket, Narayanan Sujatha, Ranganath Priya, Devi Rema

**Affiliations:** 1 *Department of Anatomy, Sri Manakula Vinayakar Medical College, Pondicherry, India*; 2 *Department of Anatomy, St. John’s Medical College, Sarjapur Road, Bangalore- 560034, Karnataka, India*

**Keywords:** Bilateral variability, Q angle, Tibial tuberosity

## Abstract

**Objective(s):**

The objective of this study was to document and explain bilateral differences in the Q angle.

**Materials and Methods:**

Two hundred limbs of healthy adult Indian volunteers were studied. The Q angle was measured using a goniometric method with the subjects supine, quadriceps relaxed and lower limbs in neutral rotation. The relative lateral placement of the tibial tuberosity with respect to the centre of the patella was measured. Appropriate statistical tests were used to determine the bilateral variability in the Q angle and the lateral placement of the tibial tuberosity. Inter-observer variation of the above mentioned parameters were studied in twenty limbs.

**Results:**

The average Q angle value of all the 200 limbs was 12.73 °C; the mean value on the right was 12.86 **°**C and 12.60** °**C on the left. When the Q angle and the lateral placement of the tibial tuberosity were considered in pairs a significant difference was noted in males. The Q angle value on the right side was more often greater than the left. The relative lateral placement of the tibial tuberosity showed a significant positive correlation with the Q angle. The intra-class correlation coefficient was 0.66 for the Q angle and 0.8 for the lateral placement of the tibial tuberosity.

**Conclusion:**

The present study shows that bilateral variability in the Q angle could be attributed to an alteration of the relative placement of the tibial tuberosity with respect to the centre of the patella.

## Introduction

The Q angle was first defined by Brattstrom (1). He described the Q angle as an angle with its apex at the patella, and formed between the ligamentum patellae and the extension of the line formed by the quadriceps femoris muscle resultant force (1). It was later measured using the anterior superior iliac spine (ASIS) as the proximal landmark (2). The Q angle provides an estimate of the vector force between the quadriceps femoris muscle and the patellar tendon (3). It is formed by the crossing of two imaginary lines. The first line extends from the ASIS to the centre of the patella (CP). The second line is drawn from the tibial tuberosity (TT) to the CP. The angle formed between these two lines represents the Q angle. The Q angle has come to be accepted as an important factor in assessing knee joint function (4). An increase in Q angle beyond the normal range is considered as indicative of extensor mechanism misalignment, and has been associated with patellofemoral pain syndrome, knee joint hypermobility and patellar instability (5-7). Moreover, its role in assessing other lower-extremity injuries in sports and military populations has been documented (8).

Though bilateral differences in the Q angle have been documented, most studies done so far have concentrated on between-group rather than within-subject differences (9-12). Moderate to substantial amounts of bilateral asymmetry in the Q angle values when analyzed on an individual basis has been demonstrated (9,13). This has been attributed to bilateral asymmetry in the quadriceps muscle strength (13). However, within-subject bilateral differences in the relative position of the CP and TT, which are likely to alter the value of the Q angle have not been demonstrated. The aims of this study were, to document bilateral differences in the mean Q angle, to note differences in the value of the Q angle between the right and left sides in an individual, to study whether there was any difference of the above findings in males and females and finally to discuss the possible explanation for the findings in an adult Indian population. 

## Materials and Methods

The subjects for the study were normal healthy adult volunteers and college students from St. John's Medical College, Bangalore, India. The procedure was explained to the subjects who then signed an informed consent form. Ethical clearance for the study was obtained from the Institutional Ethical Review Board (IERB). A total of 200 lower limbs (100 subjects consisting of 50 males and 50 females) were studied. Males and females of the age of 18 years and above were included in the study. The mean age of the subjects was 23 years (range 18-43 years). Using criteria described by Belchior *et al* (14), any subject with a history of the following conditions was excluded from the study: 1) Fracture of the lower limb, chronic knee pain, dislocation of the patella and spinal cord pathology with lower limb involvement. 2) Anterior or retro-patellar pain when performing at least two of the following activities: ascending stairs, being seated for long periods, upon squatting, kneeling or jumping. 3) Any history of surgery on the knee, clinical evidence of meniscal injury, ligamentous instability and patellar tendinitis. All measurements were taken once by a single investigator. Twenty measurements (bilaterally in ten subjects) were performed independently by another observer after one week to assess inter-observer variability. 

***Measurement of the Q angle***

**Figure 1:**
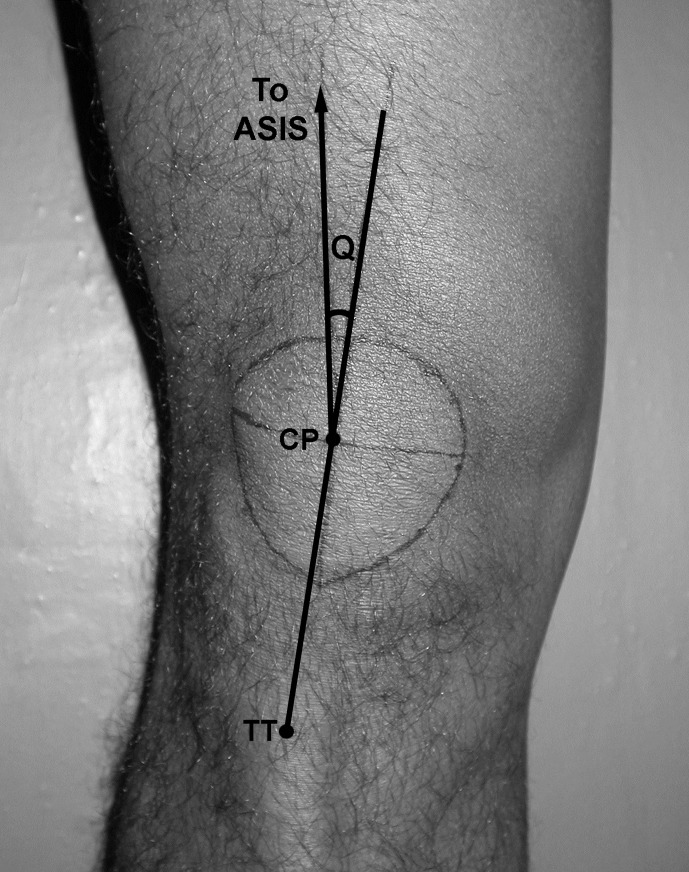
Measurement of the Q angle. ASIS – anterior superior iliac spine; CP-centre of patella; TT-tibial tuberosity; Q- quadriceps angle.

A goniometric method as described by Jha and Raza was adopted (15). The measurement of the Q angle was performed with the subject supine and keeping the pelvis square. The legs were extended at the knee joint with the quadriceps muscle relaxed. The feet were placed in a position of neutral rotation, such that the toes were pointing directly upwards and the feet were perpendicular to the resting surface*. *The following bony landmarks were marked with a marker pen: ASIS, CP and centre of the TT. The outline of the patella was drawn with a marker pen, after palpating the borders and making sure that the skin was not stretched in doing so. The CP was defined as the point of intersection of the maximum vertical and transverse diameters of the patella. The point of maximum prominence was defined as the centre of the TT. One line was drawn from the CP towards the ASIS using the straight edge of a measuring tape and represented the longitudinal axis of the femur. Another line joined the centre of the TT and the CP. The second line was extended upwards. The angle formed between the above two lines was defined as the Q angle and measured with a goniometer (Figure 1). 

**Figure 2:**
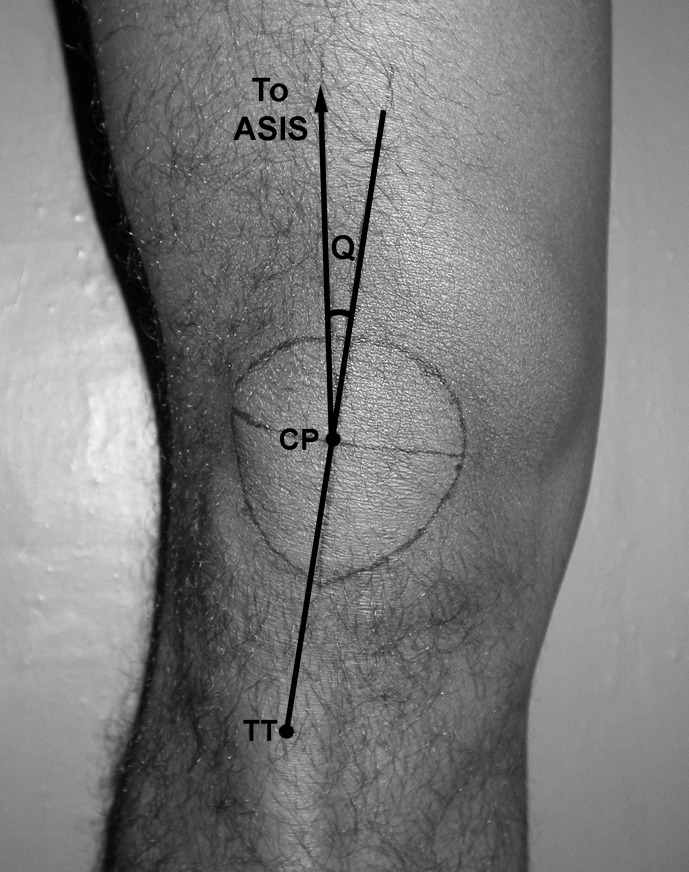
Determination of the relative lateral placement of the tibial tuberosity with respect to the centre of patella. CP -centre of patella; TT-tibial tuberosity; A-point of intersection of vertical line drawn from CP and horizontal line drawn from TT; d-lateral placement of TT

***Measurement of relative position of CP and TT***

A frontal view digital photograph of the knee with the markings mentioned above was taken with a scale and the lateral placement of the TT was calculated as follows using Adobe Photoshop software. A vertical line was drawn inferiorly from the CP. A horizontal line was drawn from the TT to meet the above line at A (Figure 2). The distance from TT to A (d in Figure 2) was measured in centimeters (to the nearest millimeter) and represented the lateral placement of the TT with respect to the CP. 

## Results

The average Q angle value of all the 200 limbs was 12.73 ºC. The mean Q angle value on the right side was 12.86 ºC as compared to 12.60 ºC on the left. The mean values of the Q angle and the lateral placement of the TT did not show significant bilateral differences. However, when the values of the Q angle were compared in pairs between the right and left side, significant bilateral variability was noted (Table 1). The Q angle showed a significant positive correlation (r= 0.49, *P*< 0.001) with the lateral placement of the TT. The inter-observer correlation coefficients for the Q angle and lateral placement of the TT were 0.66 and 0.80 respectively.

In males, the average Q angle value of the 100 limbs was 10.98 **°**C. The value on the right side was 11.24 **°**C as compared to 10.24** °**C on the left. The mean values of the Q angle did not show significant bilateral differences. However, the mean lateral placement of the TT was significantly greater on the right side. Paired comparison of the values of the Q angle and the lateral placement of the TT between the right and left side revealed significant differences (Table 1). A greater mean Q angle value of 14.48** °**C was noted in the 100 female limbs. The mean Q angle value on both right and left sides was 14.48** °**C. No bilateral differences in the mean values of the Q angle and the lateral placement of the TT were observed. When the values of the Q angle and the lateral placement of the TT were compared in pairs between the right and left side no significant bilateral variability was noted (Table 1). When the difference between the right and left Q angles was tabulated it was noted that in 36% of the subjects there was no bilateral difference. The Q angle was more often greater on the right side as compared to the left, both in males and females. However bilateral asymmetry in the values was more commonly seen in males (Table 2). 

**Table 1:** Bilateral comparison between Q angle values and placement of the tibial tuberosity d- lateral placement of tibial tuberosity; n - number of limbs studied; SD – standard deviation; * , ** - significant parameters † - Mann-Whitney U test; ‡ - Wilcoxon sign rank test

Parameter subjects(n)		Right (Mean±SD)	Left (Mean±SD)	Significance (*P* values)		

				Between groups ^†^	Within Subject^‡^	
Q - angle	All (200)	12.86 ± 2.36	12.6 ± 2.78	0.20	0.02^*^	
	Males (100)	11.24 ± 1.67	10.24 ± 2.29	0.10	0.01^*^	
	Females (100)	14.48 ± 1.76	14.48 ± 3.03	0.44	0.28	
d	All (200)	1.40 ± 0.74	1.31 ± 0.80	0.13	0.08	
	Male (100)	1.1 ± 0.46	0.82 ± 0.44	0.004^*^	0.003^**^	
	Female (100)	1.78 ± 0.64	1.86 ± 0.66	0.33	0.18	

**Table 2:** Individual differences between Q angle values on the right and left sides.

Difference between right and left Q angle in degrees	Right = Left**†**		Right >Left ^‡^		Left > Right^§^	
	Males (n = 50)	Females (n = 50)	Males (n = 50)	(Females (n = 50)	Males (n = 50)	Females (n = 50)
0	12 (24%)	24 (48%)	-	-	-	-
1	-	-	19 (38%)	11 (22%)	10 (20%)	4 (8%)
2	-	-	5 (10%)	3(6%)	2 (4%)	1(2%)
3	-	-	1 (2%)	3 (6%)	0	1 (2%)
>3	-	-	1 (2%)	0	0	3 (6%)

**†** - number of subjects with no bilateral differences in the Q angle

**‡** - number of subjects with right Q angle greater that left Q angle

**§** - number of subjects with left Q angle greater than right Q angle

**n** - number of subjects

**Table 3:** Comparison between different studies on the bilateral variability in the mean Q angle

Author	Year	Number of normal subjects studied	Bilateral variability in mean Q angle values	Method of measurement	Details
Hahn and Foldspang	1997	339	R>L	Universal goniometer	Supine position with quadriceps relaxed, and legs strapped together
Livingston and Mandigo	1997	50	L>R	Universal goniometer	Standing position with quadriceps relaxed
Byl and Livingston	2000	34	R>L	Universal goniometer	Standing position with the medial borders of the feet in contact
Livingston and Spaulding	2002	20	R>L^*^	OPTOTRAK	Standing position with quadriceps relaxed and the feet in Romberg stance
Sra *et al*	2008	70	L>R^*^	Universal goniometer	Standing position with quadriceps relaxed and the feet in Romberg stance
Present study	2009	100	R>L	Universal goniometer	Subjects supine with quadriceps relaxed and feet in neutral rotation

values R and L – right and left sides respectively

* significant differences noted

## Discussion

Though numerous studies on the Q angle have been conducted worldwide, relatively few of them have focused on its bilateral variability. Minor bilateral variations of bodily structures are a rule rather than an exception. However, significant differences warrant closer scrutiny. Hahn and Foldspang were among the first investigators to make a detailed study of the bilateral variability in the Q angle (10). Following this, other studies have documented similar bilateral variations (9, 11-13). In some of these studies it was found that the mean Q angle on the right side was greater than that on the left (10, 11,13). In other studies the mean Q angle was more on the left as compared to the right (9,12). In only two of the studies were these differences significant (11,12). In the present study the mean Q angle was greater on the right side as compared to the left but this difference was not statistically significant (Table 3).

Though minor bilateral differences in the mean Q angle could be explained as a result of normal variation or minor errors in measurement, significant differences need further explanation. One of the explanations put forth for this is the bilateral difference in the quadriceps strength. It was found that the Q angle varied inversely with the peak torque angle during active knee extension (13). However, in the studies which showed significant bilateral differences in the Q angle, the quadriceps muscle was relaxed (11,12). In one of the studies, the sample size was relatively small (20 individuals), which could be a possible explanation for the significant bilateral difference in the mean Q angle (11). 

Though many of the studies referred to above did not show significant bilateral differences in the mean Q angle, within-subject differences in the Q angle were noted in only a few studies (9,13). When studying bilateral differences in the Q angle, comparing means may give misleading results. In previous studies a difference of less than 4 °C was noted in 32% of subjects by Livingston and Mandigo (9) and in 35% of subjects by Byl *et al* (13). In the above studies the Q angle was measured with the subjects in the standing position. In the present study a difference of less than 3 °C was noted in 96% of subjects, with bilateral variability being greater in males as compared to females (Table 2). The increased variation noted in the other studies could be due to accentuation of bilateral Q angle differences due to weight bearing. The previous study done in India by Jha and Raza was done in the supine position.^15^ For accurate comparison with the previous study the authors of the present study used a similar method, keeping in mind the effect of limb position on the magnitude of the Q angle. One of the limitations of the present study is that side differences in the Q angle of the weight-bearing knee were not analyzed. An explanation for the difference in males and females could be due to greater asymmetric limb usage in males, leading to more bilateral variability in the quadriceps muscle tone in them. In the present study, though there was no significant bilateral difference in the mean Q angle value, when considered as pairs there was a significant difference noted between the right and left sides. This difference was noted when all the subjects were taken as a whole and in males, but not in females (Table 1). 

Any bilateral difference in the Q angle has to necessarily be due to a relative alteration of the three bony points used to measure it. The position of the ASIS being relatively fixed, it is unlikely to be a cause for bilateral variability. This variability can then be attributed due to a relative alteration in the positions of the CP and the TT. The relative lateral placement of the TT with respect to the CP was measured in the present study. The bilateral variability in this parameter was significantly greater in males as compared to females with a higher mean value on the right side (Table 1). The Q angle showed a significant positive correlation with the relative lateral placement of the TT. This indicates that alteration of the relative placement of the distal two bony landmarks could be a cause for bilateral variability in the Q angle. In the present study, a good degree of inter-observer correlation of 0.8 for the lateral placement of the TT with respect to the CP indicates that the method described is a reliable one. 

The accurate determination of the Q angle requires precise identification of the three bony landmarks used to measure it. France and Nester found that even small differences in the placement of the CP and TT could alter the Q angle greatly (16). There is a subjective element in determining the CP as it depends on marking of the intersection of the greatest transverse and vertical diameters. Also, the centre of the TT cannot be determined precisely in some subjects. In these subjects the TT is a plateau atop an elevation. Thus, the findings in the present study need to be validated using more accurate methods, such as those described by Roush *et al* (17). 

Some authors have questioned the reliability and validity of the Q angle in evaluating and treating patello-femoral joint pathology (18-20). Smith* et al* in a systematic review of the literature found that there is a lack of standardization in the measurement procedure of the Q angle (21). Thus, bilateral variability of the Q angle could be influenced by the procedure used to measure it. The inter-observer variability in the Q angle from different studies has varied widely from 0.17 to 0.97 (18,22). In the present study it was 0.66. The inter-tester reliability of the Q angle could be improved by proper standardization of the method, and adequate training of the testers (22). In spite of the above limitations of the present study, the authors feel that it could have some value in explaining the side differences that exist in the values of the Q angle. 

## Conclusion

The present study documents bilateral variations in the Q angle in young healthy adults. All measurements were made with the subjects supine, the quadriceps relaxed and the feet in neutral rotation. The relative lateral placement of the TT with respect to the CP was noted. Mean Q angle measurements were marginally greater on the right side when males and females were considered together. This difference was more in males, though it was not significant. Even though bilateral mean Q angle values were not significantly different, when taken in pairs a significant difference was noted in males. On tabulating the differences between the right and left Q angle values it was noted that in 96% of the subjects the value was less than 3 °C. A greater bilateral variability was noted in males as compared to females. The present study shows that this bilateral variability in the Q angle could be attributed to an alteration of the relative placement of the TT with respect to the CP on each side. Though the present study may not have any direct clinical applications, it is likely to be useful in explaining side differences in the Q angle.

## References

[1] Brattstrom H (1964). Shape of the intercondylar groove normally and in recurrent dislocation of patella. Acta Orthop Scand Suppl.

[2] Insall J, Falvo DA, Wise DW (1976). Chondromalacia patellae: A prospective study. J Bone Joint Surg[Am].

[3] Livingston LA (1998). The quadriceps angle: A review of the literature. J Orthop Sports Phys Ther.

[4] Emami MJ, Ghahramani MH, Abdinejad F, Namazi H (2007). Q-angle: An invaluable parameter for evaluation of anterior knee pain. Arch Iran Med.

[5] Waryasz GR, McDermott AY (2008). Patellofemoral pain syndrome (PFPS): a systematic review of anatomy and potential risk factors. Dyn Med.

[6] Sendur OF, Gurer G, Yildirim T, Ozturk E, Aydeniz A (2006). Relationship of Q angle and joint hypermobility and Q angles in different positions. Clin Rheumatol.

[7] Smith TO, Davies L, OrsquoDriscoll ML, Donell ST (2008). An evaluation of the clinical tests and outcome measures used to assess patellar instability. Knee.

[8] Rauh MJ, Koespsell TD, Rivara FP, Rice SG, Margherita AJ (2007). Quadriceps angle and risk of injury among high school cross country runners. J Orthop Sports Phys Ther.

[9] Livingston LA, Mandigo JL (1997). Bilateral within-subject Q angle asymmetry in young adult females and males. Biomed Sci Instrum.

[10] Hahn T, Foldspang A (1997). The Q-angle and sport. Scand J Med Sci Sports.

[11] Livingston LA, Spaulding SJ (2002). OPTOTRAK Measurement of the Quadriceps angle using standardized foot positions. J Athl Train.

[12] Sra A, Ba T, Oo J (2008). Comparison of bilateral Quadriceps angle in asymptomatic and symptomatic males with anterior knee pain. Internet J Pain Symptom Contr Palliative Care.

[13] Byl T, Cole JA, Livingston LA (2000). What determines the magnitude of the Q angle? A preliminary study of selected skeletal and muscular measures. J Sport Rehabil.

[14] Belchior ACG, Arakaki JC, Bevilaqua-grossi D, Reis FA, Carvalho PTC (2006). Effects on the Q angle measurement with maximal isometric contraction of the quadriceps muscle. Rev Bras Med Esporte.

[15] Jha A, Raza HKT (2000). Variation in Q-angle according to sex, height, weight and interspinous distance - A Survey. Int J Orthod.

[16] France L, Nester C (2001). Effect of errors in the identification of anatomical landmarks on the accuracy of Q angle values. Clin Biomech.

[17] Roush JR, Bustillo K, Low E (2008). Measurement error between a goniometer and the NIH image J program for measuring quadriceps angle. Internet J of Allied Health Sci Pract.

[18] Greene CC, Edwards TB, Wade MR, Carson EW (2001). Reliability of the Quadriceps angle measurement. Am J Knee Surg.

[19] Sanfridsson J (2001). Orthopaedic measurements with computed radiography: methodological development, accuracy, and radiation dose with special reference to the weight-bearing lower extremity and the dislocating patella. Acta Radiol.

[20] Smith TO, Davies L, OrsquoDriscoll ML, Donell ST (2008). An evaluation of the clinical tests and outcome measures used to assess patellar instability. Knee.

[21] Smith TO, Hunt NJ, Donell ST (2008). The reliability and the validity of the Q angle: a systematic review. Knee Surg Sports Traumatol Arthrosc.

[22] Shultz SJ, Ngyyen AD, Windley TC, Kulas AS, Botic TL, Beynnon BD (2006). Intratester and intertester reliability of clinical measures of lower extremity anatomic characteristics: implications for multicenter studies. Clin J Sport Med.

